# Prevalence of Poor Sleep Quality and Associated Factors Among Medical Students in Saudi Arabia

**DOI:** 10.7759/cureus.114806

**Published:** 2026-08-19

**Authors:** Saeed A Alghamdi, Mohammed S Alameri, Anas I Ibn Auf

**Affiliations:** 1 Psychiatry, Erada and Mental Health Complex, Taif, SAU; 2 Psychiatry, Eastern Sudan University for Medical Sciences and Technology, Port Sudan, SDN

**Keywords:** cross-sectional study, kessler k10, late-night device use, medical students, pittsburgh sleep quality index, psychological distress, saudi arabia, screen time, sleep hygiene, sleep quality

## Abstract

Background: Sleep disturbances are common among medical students, whose demanding academic environment combines heavy workloads, clinical rotations, and frequent examinations. This study examined the prevalence of poor sleep quality and its association with psychological distress, lifestyle factors, and electronic-device use (e.g., smartphones, tablets, and laptops) among medical students in Saudi Arabia.

Methods: In this cross-sectional study, 215 medical students from multiple Saudi universities completed an online self-administered questionnaire comprising sociodemographic, social, medical, and psychiatric items, the Pittsburgh Sleep Quality Index (PSQI), the Kessler Psychological Distress Scale (K10), and questions on electronic-device and screen use. Poor sleep quality was defined as a global PSQI score of >5. Associations were assessed using chi-squared and Fisher's exact tests, independent samples t-tests, Mann-Whitney U tests, and Spearman's rank correlation, with p < 0.05 considered significant.

Results: Poor sleep quality was highly prevalent, affecting 79.1% of participants. Most sociodemographic and lifestyle factors, including age, gender, academic year, smoking, body mass index, and caffeine intake, were not significantly associated with sleep quality. Marital status reached significance (p = 0.043) but was limited by the very small number of married or divorced participants. Late-night device use was significantly associated with poor sleep (p = 0.046). Psychological distress was common (mean K10: 25.87 ± 10.56), with more than half of the students (51.2%) reporting moderate-to-severe distress. Poor sleepers had significantly higher K10 scores than good sleepers (27.65 ± 10.06 vs. 19.13 ± 9.73; p < 0.001) and nearly sixfold higher odds of moderate-to-severe distress (odds ratio: 5.86; 95% CI: 2.65-12.93), and PSQI scores correlated positively with K10 scores (ρ = 0.442; p < 0.001).

Conclusions: Poor sleep quality is highly prevalent among medical students in Saudi Arabia and is associated with greater psychological distress and late-night device use. Structured sleep hygiene programs, guidance on night-time device use, and routine screening for psychological distress are warranted. The cross-sectional design precludes causal inference.

## Introduction

Medical students train in a highly demanding academic environment that combines intense workloads, clinical rotations, and frequent examinations. Success in this setting requires sustained cognitive performance, yet the same pressures frequently deprive students of adequate, good-quality sleep. Sleep disturbances, including insomnia symptoms in up to a third of students and short sleep durations averaging fewer than six hours per night, have been widely reported in this population [1].

Sleep is essential for core cognitive processes such as attention, memory consolidation, and decision-making, and sleep deprivation degrades these functions, producing attention lapses, reduced memory retention, and impaired critical thinking [2,3]. For medical students, who rely heavily on these abilities for examinations and clinical practice, the consequences of poor sleep may be especially significant.

Lifestyle and behavioral factors are also relevant. Distress and anxiety arising from rigorous schedules may further disturb sleep, while behaviors such as high screen exposure have been linked to shorter sleep duration; one study of medical students reported an average of seven hours of screen use per day [4].

Poor sleep quality is both common and consequential among medical students. A meta-analysis of observational studies found it to be highly prevalent across a range of countries and training systems [5]. Beyond its prevalence, poor sleep has been linked to impaired academic performance [1] and is closely associated with burnout and reduced quality of life during medical training [6].

Despite the recognized burden of sleep disturbance among medical students, research examining its associated psychological, lifestyle, and behavioral factors in Saudi medical students remains limited. The present study therefore aimed to determine the prevalence of poor sleep quality among medical students in Saudi Arabia and to examine its association with psychological distress, lifestyle factors, and electronic-device use.

## Materials and methods

Study design and setting

A cross-sectional, non-interventional study was conducted among medical students enrolled at universities in Saudi Arabia between 27/09/2024 and 07/07/2025. Participants were recruited through an online survey administered using Google Forms (Google LLC, Mountain View, CA, USA) and distributed primarily via student channels at King Saud bin Abdulaziz University for Health Sciences in Jeddah and Taif University in Taif, Saudi Arabia; students from other Saudi medical schools could also participate. A single central ethical approval from the Scientific Research Ethics Committee at King Faisal Medical Complex (Taif Health Cluster) (approval number: 2024-E-60), was obtained for the study; given the anonymous, voluntary, and online nature of the survey, separate institutional approvals at each university were not required.

Participants

Medical students were eligible for inclusion, and non-medical students were excluded. Participants were recruited through convenience sampling. The minimum required sample size was estimated using the single-population proportion formula, assuming an anticipated prevalence of poor sleep quality of 75% based on previous studies among medical students [7,8], a 95% confidence level, and a 6% margin of error, which yielded a minimum of approximately 200 participants. The final sample of 215 students exceeded this minimum.

Data collection

Data were collected through a structured, self-administered online questionnaire organized into five sections: (1) sociodemographic data; (2) social, medical, and psychiatric history; (3) the Pittsburgh Sleep Quality Index (PSQI); (4) the Kessler Psychological Distress Scale (K10); and (5) items on electronic-device and screen use. Students were invited to participate through an online link distributed via university channels and student groups, and informed consent was obtained electronically before participation. Participation was voluntary and anonymous. Because the total number of students reached by the invitation could not be determined, a response rate could not be calculated.

Measures

Sleep quality was assessed with the PSQI, a validated self-report instrument whose global score ranges from 0 to 21; a global score greater than 5 identifies poor sleepers [9]. An Arabic-validated version of the PSQI was administered [10]. Psychological distress was measured with the K10, a validated 10-item measure of non-specific distress over the previous 30 days, with higher scores indicating greater distress [11]. K10 total scores range from 10 to 50 and were categorized using the standard severity bands: 10-19 (likely well), 20-24 (mild distress), 25-29 (moderate distress), and 30-50 (severe distress). An Arabic-validated version of the K10 was administered [12]. Electronic-device use was captured through self-reported daily device-use duration (in hours), late-night device use (defined as any device use after 8 p.m.), and device use in the hour immediately before sleep. The 8 p.m. threshold was selected specifically for this study to capture device use during the evening and pre-sleep period and was not derived from prior literature.

Statistical analysis

Data were analyzed using IBM SPSS Statistics for Windows, V. 21.0 (IBM Corp., Armonk, NY, USA). Categorical variables were summarized as frequencies and percentages and continuous variables as mean ± standard deviation. Associations between categorical variables and sleep-quality category were examined using the chi-squared test, with Fisher's exact test where expected cell counts were below 5. Continuous variables were compared between good and poor sleepers using independent samples t-tests. Because K10 scores were not normally distributed (Shapiro-Wilk test; p < 0.05), psychological distress scores were compared between good and poor sleepers using the Mann-Whitney U test, with medians reported alongside means. The distribution of K10 severity categories across sleep-quality groups and the proportion of students with moderate-to-severe distress (K10 ≥25) were examined using the chi-squared test, and an odds ratio with a 95% confidence interval was calculated for moderate-to-severe distress. The association between PSQI and K10 total scores was assessed using Spearman's rank-order correlation. Because the first four PSQI items were collected as free-text responses, answers given as ranges were converted to their midpoints (e.g., "1-2 hours" was scored as 1.5 hours; "6-8 a.m." as 7 a.m.), and approximate descriptors were mapped to representative clock times before scoring according to the standard PSQI algorithm. Responses that could not be quantified (e.g., "varies greatly") were treated as missing; as a result, 23 participants lacked one or more component scores, most commonly the sleep-efficiency component. To retain the full sample, these missing components were replaced with the median of the available responses for the corresponding component, and the resulting good/poor classification was identical across a range of imputation approaches (sensitivity analysis); accordingly, the good/poor classification, the comparison of K10 scores between sleep-quality groups, and the PSQI-K10 correlation used the full sample of 215. A two-sided p-value below 0.05 was considered statistically significant.

## Results

Baseline characteristics

Out of 215 medical students included in this study, the mean age was 21.8 ± 1.8 years, ranging from 18 to 30 years. Male participants predominated (73.5%), and 99.1% were single. In terms of academic year, 61.9% were in the clinical years (years 4-6), 35.8% in the preclinical phase (years 1-3), and 2.3% in their internship year (year 7). Participants were recruited from multiple institutions, most commonly King Saud bin Abdulaziz University for Health Sciences (40.9%) and Taif University (21.4%), with the remainder from other Saudi medical schools. Regarding lifestyle factors, normal or underweight BMI was recorded in 61.2%, while 24.8% were overweight, and 14.0% were classified as obese. Only 13.0% reported having at least one medical condition, and 14.9% self-reported having a psychiatric disorder. Smoking was relatively uncommon (9.3%).

Caffeine consumption was highly prevalent, with 89.8% reporting regular intake of at least one caffeinated beverage. Tea was the most consumed, with 73% reporting regular intake, followed by coffee at 69.8%, while 27.9% consumed energy drinks. Occasional consumption (less than daily) was the most common tea-consumption pattern, reported by 41.4% of participants, followed by one to two servings daily (16.3%). For coffee, occasional consumption (less than one cup per day) was the most common pattern, reported by 36.2% of participants, followed by one to two cups daily (24.6%). For energy drinks, 19.8% of participants reported occasional consumption (less than one per day), while daily consumption was rare. Baseline characteristics are presented in Table 1.

**Table 1:** Baseline characteristics of the study participants (n = 215) BMI could not be computed for one participant with implausible height/weight values (n = 214).

Characteristic	n (%) or mean ± SD
Age (years)	21.8 ± 1.8 (range: 18-30)
Gender	
Male	158 (73.5)
Female	57 (26.5)
Marital status	
Single	213 (99.1)
Married/divorced	2 (0.9)
Study stage	
Preclinical (years 1-3)	77 (35.8)
Clinical (years 4-6)	133 (61.9)
Internship (year 7)	5 (2.3)
University	
King Saud bin Abdulaziz University for Health Sciences	88 (40.9)
Taif University	46 (21.4)
Other/unspecified	81 (37.7)
BMI	
Underweight/normal (<25.0 kg/m²)	131 (61.2)
Overweight (25.0-29.9 kg/m²)	53 (24.8)
Obesity (≥30.0 kg/m²)	30 (14.0)
Smoking	
Yes	20 (9.3)
No	195 (90.7)
Caffeinated drinks (any)	
Yes	193 (89.8)
No	22 (10.2)
Tea consumption	
Yes	157 (73.0)
No	58 (27.0)
Coffee consumption	
Yes	150 (69.8)
No	65 (30.2)
Energy drink consumption	
Yes	60 (27.9)
No	155 (72.1)
Any medical condition	
Yes	28 (13.0)
No	187 (87.0)
Any psychiatric disorder (self-reported)	
Yes	32 (14.9)
No	183 (85.1)

Prevalence of poor sleep quality

Sleep quality was assessed using the PSQI, with a validated cutoff score of >5 indicating poor sleep quality. A high prevalence of sleep disturbance was observed among medical students. Of the 215 participants, 79.1% had poor sleep quality, while 20.9% had good sleep quality, indicating that four out of every five medical students experienced sleep difficulties.

Table 2 presents the associations of sociodemographic, lifestyle, and clinical characteristics with sleep quality. Marital status showed a statistically significant association with sleep quality (p = 0.043); however, this finding should be interpreted with caution, as only two participants were married or divorced and all poor sleepers were single. Age was comparable between good and poor sleepers (22.07 ± 1.84 vs. 21.74 ± 1.74 years, respectively; p = 0.271), and males were predominant in both groups. Clinical years (years 4-6) represented the largest proportion of participants in both sleep-quality groups, comprising 66.7% of good sleepers and 60.6% of poor sleepers.

**Table 2:** Comparison of sociodemographic, lifestyle, and clinical characteristics by sleep quality (PSQI) Data are presented as mean ± standard deviation for continuous variables and number (percentage within sleep-quality category) for categorical variables. Percentages are calculated based on valid cases; therefore, totals may not sum to the overall sample size due to missing data. An independent samples t-test was used for age and a chi-squared test for categorical variables, with Fisher's exact test where any expected cell count was <5; exact tests yield no test statistic (indicated by -). Statistical significance was defined as p < 0.05. BMI could not be computed for one participant with implausible height/weight values (n = 214). PSQI: Pittsburgh Sleep Quality Index

Variables	Category	Good sleep, n (%)	Poor sleep, n (%)	Test statistic (df)	P-value
Age	Mean ± SD	22.07 ± 1.84	21.74 ± 1.74	t = 1.10 (df = 213)	0.271
Gender	Male	35 (77.8)	123 (72.4)	χ² = 0.54 (df = 1)	0.463
	Female	10 (22.2)	47 (27.6)		
Marital status	Single	43 (95.6)	170 (100.0)	-	0.043
	Married/divorced	2 (4.4)	0 (0.0)		
Study stage	Preclinical (years 1-3)	13 (28.9)	64 (37.6)	χ² = 2.07 (df = 2)	0.355
	Clinical (years 4-6)	30 (66.7)	103 (60.6)		
	Internship (year 7)	2 (4.4)	3 (1.8)		
Smoking	No	40 (88.9)	155 (91.2)	-	0.576
	Yes	5 (11.1)	15 (8.8)		
BMI	Underweight/normal	26 (57.8)	105 (62.1)	χ² = 0.29 (df = 2)	0.865
	Overweight	12 (26.7)	41 (24.3)		
	Obesity	7 (15.6)	23 (13.6)		
Any caffeinated drink	No	3 (6.7)	17 (10.0)	-	0.575
	Yes	42 (93.3)	153 (90.0)		
Tea consumption	No	12 (26.7)	46 (27.1)	χ² = 0.003 (df = 1)	0.958
	Yes	33 (73.3)	124 (72.9)		
Coffee consumption	No	11 (24.4)	54 (31.8)	χ² = 0.90 (df = 1)	0.342
	Yes	34 (75.6)	116 (68.2)		
Energy drinks	No	33 (73.3)	122 (71.8)	χ² = 0.04 (df = 1)	0.835
	Yes	12 (26.7)	48 (28.2)		
Any medical illness	No	40 (88.9)	147 (86.5)	χ² = 0.18 (df = 1)	0.668
	Yes	5 (11.1)	23 (13.5)		
Any mental disorder	No	39 (86.7)	144 (84.7)	χ² = 0.11 (df = 1)	0.742
	Yes	6 (13.3)	26 (15.3)		
All participants		45 (20.9)	170 (79.1)		

Lifestyle factors and sleep quality

Regarding lifestyle factors, most participants in both groups were non-smokers, with 88.9% of good sleepers and 91.2% of poor sleepers. Underweight/normal weight was observed in 57.8% of good sleepers and 62.1% of poor sleepers. Notably, caffeine consumption was highly prevalent in both groups, with 93.3% of good sleepers and 90.0% of poor sleepers consuming caffeine. Most participants in both groups reported no medical illnesses (88.9% of good sleepers vs. 86.5% of poor sleepers) and no mental disorders (86.7% of good sleepers vs. 84.7% of poor sleepers).

Beverage consumption frequency patterns also showed no statistically significant associations with sleep quality for any of the caffeinated beverages examined (Table 3). The most common tea-consumption pattern in both groups was occasional consumption (less than daily), reported by 51.1% of good sleepers and 38.6% of poor sleepers. For coffee consumption, occasional consumption (less than one cup per day) was also the predominant pattern among good sleepers (51.1%), while poor sleepers showed more varied consumption across categories. Energy drink consumption was notably infrequent overall, with 76.7% of each group reporting no consumption.

**Table 3:** Detailed analysis of caffeinated beverage consumption patterns and sleep quality (PSQI) P-values are from chi-squared tests. Statistical significance was defined as p < 0.05. Missing data: tea (n = 12); coffee (n = 8); energy drinks (n = 13). PSQI: Pittsburgh Sleep Quality Index

Beverage/frequency	Overall, n (%)	Good sleep (PSQI ≤5), n (%)	Poor sleep (PSQI >5), n (%)	Test statistic (df)	P-value
Tea consumption					
None/not applicable	58 (28.6)	12 (26.7)	46 (29.1)	χ² = 2.71 (df = 3)	0.439
Occasional (less than daily)	84 (41.4)	23 (51.1)	61 (38.6)		
1-2 per day	33 (16.3)	6 (13.3)	27 (17.1)		
≥3 per day	28 (13.8)	4 (8.9)	24 (15.2)		
Coffee consumption					
None/not applicable	65 (31.4)	11 (24.4)	54 (33.3)	χ² = 5.58 (df = 3)	0.134
Occasional (less than daily)	75 (36.2)	23 (51.1)	52 (32.1)		
1-2 per day	51 (24.6)	8 (17.8)	43 (26.5)		
≥3 per day	16 (7.7)	3 (6.7)	13 (8.0)		
Energy drink consumption					
None/not applicable	155 (76.7)	33 (76.7)	122 (76.7)	χ² = 2.22 (df = 3)	0.528
Occasional (less than daily)	40 (19.8)	10 (23.3)	30 (18.9)		
1-2 per day	4 (2.0)	0 (0.0)	4 (2.5)		
≥3 per day	3 (1.5)	0 (0.0)	3 (1.9)		

Electronic-device use and sleep quality

The relationship between characteristics of electronic-device use and sleep quality was examined. Device usage patterns revealed that late-night device use was significantly associated with poor sleep quality (p = 0.046); however, the absolute difference between groups was small, as late-night device use was reported by 95.6% of good sleepers and 100.0% of poor sleepers.

Daily device-use duration was higher among poor sleepers (8.47 ± 3.21 hours) compared to good sleepers (7.64 ± 3.08 hours), although this difference did not reach statistical significance (p = 0.130). Similarly, device use immediately before sleep was nearly universal in both groups, reported by 95.6% of good sleepers and 99.4% of poor sleepers, with no significant difference between groups (p = 0.120). All device-use comparisons are presented in Table 4.

**Table 4:** Association between electronic-device use characteristics and sleep quality (PSQI) Data are presented as mean ± SD for continuous variables and number (percentage within sleep category) for categorical variables. *Independent samples t-test (equal variances assumed; Levene's test p = 0.676). **Fisher's exact test; exact tests yield no test statistic (indicated by -). Statistical significance was defined as p < 0.05. Missing data: daily device-use duration (n = 9); late-night device use (n = 8); device use immediately before sleep (n = 8). PSQI: Pittsburgh Sleep Quality Index

Variable	Category	Good sleep (PSQI ≤5), n (%) or mean ± SD	Poor sleep (PSQI >5), n (%) or mean ± SD	Test statistic (df)	P-value
Daily device-use duration (hours)	Mean ± SD	7.64 ± 3.08	8.47 ± 3.21	t = -1.52 (df = 204)	0.130*
Late-night device use	No	2 (4.4)	0 (0.0)	-	0.046**
	Yes	43 (95.6)	162 (100.0)		
Device use immediately before sleep	No	2 (4.4)	1 (0.6)	-	0.120**
	Yes	43 (95.6)	161 (99.4)		

Psychological distress and sleep quality

Psychological distress among medical students, as measured by the K10, revealed notable patterns. The mean total score was 25.87 ± 10.56 (median: 25; IQR: 16), indicating that students experienced moderate-to-high levels of psychological distress. When categorized using the standard K10 severity bands, 65 students (30.2%) were classified as likely well (scores 10-19), 40 (18.6%) as mild distress (20-24), 39 (18.1%) as moderate distress (25-29), and 71 (33.0%) as severe distress (30-50); overall, more than half of the participants (51.2%) reported moderate-to-severe psychological distress.

Psychological distress differed markedly by sleep quality (Table 5). Poor sleepers had significantly higher K10 scores than good sleepers (27.65 ± 10.06 (median: 26) vs. 19.13 ± 9.73 (median: 17); Mann-Whitney U test = 1857.5; p < 0.001). The distribution of K10 severity categories also differed significantly between the two groups (χ² = 33.46; df = 3; p < 0.001): severe distress was reported by 37.6% of poor sleepers compared with 15.6% of good sleepers, whereas 64.4% of good sleepers scored in the likely well range compared with 21.2% of poor sleepers. Moderate-to-severe distress (K10 ≥25) was present in 59.4% of poor sleepers versus 20.0% of good sleepers (χ² = 20.57; p < 0.001), corresponding to nearly sixfold higher odds among poor sleepers (odds ratio: 5.86; 95% CI: 2.65-12.93).

**Table 5:** Psychological distress (K10) by sleep quality (PSQI) Percentages are calculated within sleep-quality groups. *Mann-Whitney U test. **Chi-squared test. Statistical significance was defined as p < 0.05. A global PSQI score was available for all 215 participants (45 good sleepers and 170 poor sleepers), all of whom completed the K10. PSQI: Pittsburgh Sleep Quality Index; K10: Kessler Psychological Distress Scale

Variable	Good sleep (PSQI ≤5)	Poor sleep (PSQI >5)	Test statistic (df)	P-value
K10 total score, mean ± SD (median)	19.13 ± 9.73 (17)	27.65 ± 10.06 (26)	U = 1857.5	<0.001*
K10 severity category, n (%)				
Likely well (10-19)	29 (64.4)	36 (21.2)	χ² = 33.46 (df = 3)	<0.001**
Mild distress (20-24)	7 (15.6)	33 (19.4)		
Moderate distress (25-29)	2 (4.4)	37 (21.8)		
Severe distress (30-50)	7 (15.6)	64 (37.6)		
Moderate-to-severe distress (K10 ≥25), n (%)	9 (20.0)	101 (59.4)	χ² = 20.57 (df = 1)	<0.001**

A Spearman's rank-order correlation was conducted to examine the relationship between sleep quality (PSQI) and psychological distress (K10) among all 215 medical students. There was a moderate positive correlation between the two variables, which was statistically significant (ρ = 0.442; p < 0.001; n = 215). Poorer sleep quality was associated with higher levels of psychological distress.

## Discussion

This study aimed to determine the prevalence of poor sleep quality among medical students in Saudi Arabia and to explore its relationship with psychological distress, lifestyle choices, and use of electronic devices. Medical students are especially prone to sleep problems because of the intense demands of their education, heavy study loads, clinical duties, and high levels of psychological strain [13].

The most striking result was that 79.1% of the participants scored above the PSQI cutoff (>5), indicating poor sleep. That figure matches what has been reported elsewhere. For instance, Verma and colleagues found that about 76.8% of medical students in India slept poorly [14], and another cross‑sectional study of college students in China reported a prevalence of 67.9% [15]. Within Saudi Arabia, similar numbers were recorded, such as 70.4-74.2% [7,16]. Taken together, these data show that poor sleep is very common among medical students worldwide, likely because of long study hours, irregular routines, exam‑related stress, and high-performance expectations.

Most of the sociodemographic and lifestyle factors we examined (age, gender, year of study, smoking, body mass index, caffeine intake, and self‑reported medical or psychiatric history) did not show a statistically significant link to sleep quality. The average age of good sleepers (22.07 ± 1.84 years) was similar to that of poor sleepers (21.74 ± 1.74 years; p = 0.271), and men made up the majority in both groups. Clinical‑year students formed the largest share of both good (66.7%) and poor (60.6%) sleepers, but the difference was not significant (p = 0.355). So, contrary to what one might assume, being in clinical training did not reliably predict worse sleep in this sample. The lack of significant associations for these variables could be due to the modest sample size, the relatively uniform nature of the medical student population, or biases in self‑reported information.

Marital status did show a statistically significant association with sleep quality (p = 0.043). However, that finding should be interpreted with caution, as only two participants (0.9%) were married or divorced. All poor sleepers were single, but the extremely small number of non‑single individuals makes it impossible to draw any meaningful conclusions or infer causality.

Caffeine use was widespread: 89.8% of participants reported regularly having at least one caffeinated drink. Tea (73.0%) and coffee (69.8%) were the most frequently chosen. Notably, neither total caffeine intake nor the frequency of tea, coffee, or energy drink consumption was significantly associated with sleep quality. Most participants in both sleep‑quality groups consumed caffeine only occasionally (less than daily). These findings suggest that caffeine use, although common, may not be a major contributor to the high rate of poor sleep observed here; however, because caffeine dose and timing were not directly assessed, a contribution cannot be excluded. Other factors, such as academic stress and study routines, may be more important.

Using electronic devices late at night was significantly associated with poor sleep quality (p = 0.046). All poor sleepers with available data (100.0%) reported doing so, compared with 95.6% of good sleepers. Although the difference was statistically significant, the absolute gap was small (4.4 percentage points), which may limit its practical importance. Still, the near‑universal nature of late‑night device use even among those who slept well suggests that this behavior is a modifiable target for universal sleep hygiene interventions. As shown in prior research, greater screen time has been associated with delayed sleep onset and shorter sleep duration [17], while device use at bedtime has been associated with poorer sleep quality among healthcare students [18]. Total daily device use and use in the hour before sleep were not significantly associated with sleep quality in this study, even though the latter was reported by almost the same students as late-night use.

Psychological distress was common, with a mean K10 score of 25.87 ± 10.56 (median: 25; IQR: 16), indicating moderate‑to‑severe distress. There was a moderate, positive, and statistically significant correlation between poor sleep quality and higher distress (ρ = 0.442; p < 0.001; n = 215). This matches earlier work among university students [19,20], including studies done in Saudi Arabia [8,21,22]. Prior literature suggests this relationship is likely bidirectional, with poor sleep increasing vulnerability to stress and psychological distress in turn worsening sleep [13]; however, the present cross-sectional design cannot establish the temporal direction of this association.

At the group level, poor sleepers reported substantially higher distress and markedly greater odds of moderate-to-severe symptoms than good sleepers. Although the cross-sectional design cannot establish directionality, the magnitude of this difference identifies a substantial subgroup of students who may benefit from interventions that address sleep and psychological distress together.

Limitations

This study has several limitations. Its cross-sectional design precludes causal inference, so all associations are correlational. Although the sample exceeded the calculated minimum, its size may have limited power to detect small associations and to support subgroup analyses. The sample was predominantly male (73.5%), which may limit generalizability to female medical students, and participants were recruited from only a few universities, so the findings may not extend to other institutions or regions. Reliance on self-reported questionnaires introduces potential recall and social desirability bias, and sleep quality was assessed subjectively with the PSQI rather than by objective methods such as actigraphy or polysomnography. The 8 p.m. threshold used to define late-night device use captures relatively early evening exposure, which may partly explain its near-universal prevalence. The very small number of married or divorced participants (n = 2) limits the interpretation of the marital status finding, and potential confounders such as academic workload, environmental sleep conditions, and specific sleep hygiene practices were not fully assessed.

Recommendations

Even with those limitations, the findings suggest several potential implications for medical education and student well-being that warrant evaluation in future practice rather than being established by this cross-sectional study. Medical schools may consider structured sleep hygiene programs, educating students about why adequate sleep matters, and offering evidence-based strategies to improve it. Because late-night device use was nearly universal even among students who reported good sleep, campaigns encouraging less screen time before bed may be most useful when targeting the entire student body, not just those with poor sleep. Similarly, routine screening for psychological distress, combined with accessible counseling and mental health support, could help address the link between poor sleep and emotional well-being, and the high proportion of poor sleepers with moderate-to-severe distress observed here suggests that such screening could be usefully paired with questions about sleep. Future research should use longitudinal designs to clarify the direction of these associations and should include larger, more diverse samples from multiple institutions with balanced numbers of men and women.

## Conclusions

In this cross-sectional study, poor sleep quality was highly prevalent among medical students in Saudi Arabia, affecting four in five participants, and was significantly associated with greater psychological distress and with late-night electronic-device use. Poor sleepers had markedly higher distress scores and nearly sixfold higher odds of moderate-to-severe distress than good sleepers, while sociodemographic and lifestyle factors were largely not associated with sleep quality. Although the cross-sectional design precludes causal inference, the findings highlight the need for structured sleep hygiene education, guidance on night-time device use, and routine screening for psychological distress within medical training. Longitudinal, multi-institutional studies with more balanced samples are needed to clarify the direction of these associations.
